# Risk factors for stillbirths: how much can a responsive health system prevent?

**DOI:** 10.1186/s12884-018-1660-1

**Published:** 2018-01-18

**Authors:** Sutapa Bandyopadhyay Neogi, Jyoti Sharma, Preeti Negandhi, Monika Chauhan, Siddharth Reddy, Ghanashyam Sethy

**Affiliations:** 1 Indian Institute of Public Health- Delhi, Public Health Foundation of India, Gurugram, India; 2 UNICEF, Bihar, India

**Keywords:** Stillbirths, Risk factors, Health systems, Low and middle income countries

## Abstract

**Background:**

The stillbirth rate is an indicator of quality of care during pregnancy and delivery. Good quality care is supported by a functional heath system. The objective of this study was to explore the risk factors for stillbirths, particularly those related to a health system.

**Methods:**

This case-control study was conducted in two districts of Bihar, India. Information on cases (stillbirths) were obtained from facilities as reported by Health Management Information System; controls were consecutive live births from the same population as cases. Data were collected from 400 cases and 800 controls. The risk factors were compared using a hierarchical approach and expressed as odds ratio, attributable fractions and population attributable fractions.

**Results:**

Of all the factors studied, 22 risk factors were independently associated with stillbirths. Health system-related factors were: administration of two or more doses of oxytocics to augment labour before reaching the facilities (OR 1.6; 95% CI 1.2–2.1), any complications during labour (OR 2.3;1.7–3.1), >30 min to reach a facility from home (OR 1.4;1.05–1.8), >10 min to attend to the pregnant woman after reaching the facility (OR 2.8;1.7–4.5). In the final regression model, modifiable health system-related risk factors included: >10 min taken to attend to women after they reach the facilities (AOR 3.6; 95% CI 2.5–5.1), untreated hypertension during pregnancy (AOR 2.9; 95% CI 1.5–5.6) and presence of any complication during labour, warranting treatment (AOR 1.7; 95% CI 1.2–2.4). Among mothers who reported complications during labour, time taken to reach the facility was significantly different between stillbirths and live births (2nd delay; 33.5 min v/s 25 min; *p* < 0.001). Attributable fraction for any complication during labour was 0.56 (95% CI 0.42–0.67), >30 min to reach the facility 0.48 (95% CI 0.31–0.60) and institution of management 10 min after reaching the facility 0.68 (95% CI 0.58–0.75). Reaching a facility within 30 min, initiation of management within 10 min of reaching the facility and timely management of complications during labour could have prevented 17%, 37% and 20% of stillbirths respectively.

**Conclusion:**

A pro-active health system with accessible, timely and quality obstetric services can prevent a considerable proportion of stillbirths in low and middle income countries.

**Electronic supplementary material:**

The online version of this article (10.1186/s12884-018-1660-1) contains supplementary material, which is available to authorized users.

## Background

Stillbirth is an unfavourable outcome of pregnancy, which is still prevalent in many countries despite remarkable efforts to improve the care of pregnant women. Around 2.6 million third trimester stillbirths occur worldwide, out of which nearly 98% are reported from low and middle income countries (LMIC) [1]. The stillbirth rate (SBR) in India is 4/1000 births [2]. These rates are likely to be underestimates, as data on stillbirths are difficult to capture [2, 3].

Several risk factors for stillbirths have so far been documented. Maternal factors, such as advanced maternal age, teenage pregnancies, maternal nutritional status, history of prior pregnancy losses, complicated pregnancies [4] and multiple pregnancies increase the risk of stillbirths. Poor socio-economic conditions have also been found to be associated with stillbirths [5–9]. In LMICs, quality of health care is an important risk factor for stillbirth, alongside other factors. Lack of access to antenatal care services, quality of care during childbirth and delayed caesarean sections are common risk factors [10].

In India, most data on stillbirths are from hospital-based studies. Population-based data are not collected routinely and those collected are subjected to gross under-reporting and selection bias [11]. Bihar is a high-priority Indian state with a significant burden of maternal and child mortality. The routine health data collected through the Health Management Information System (HMIS) collates information from health facilities across the country. In 2015, the HMIS reported an SBR of approximately 2% for institutional deliveries in the state [12]. However, the Sample Registration System (SRS), a geographically representative population-based system of collecting data on vital statistics, reported the SBR in Bihar as 1 per 1000 births in 2015 [2].

Studies conducted in LMICs suggest that a sizeable proportion of stillbirths may have occurred during the intra-partum period, indicating that improved quality of care during childbirth and caesarean section can help prevent these stillbirths [13]. Moreover, there are anecdotal reports from certain parts of India on the administration of unwarranted use of oxytocics to augment labour, even in home deliveries [14, 15]. Studies have shown that oxytocin is used in most instances while some also administer misoprostol [16, 17]. A small pilot study conducted by the same group of authors in a neighbouring district of the same state found that nearly 20% of the women received more than two doses of such medicines to augment labour before reaching the health facilities for delivery.

For this study, the objective was to investigate the association of potential risk factors including various socio-demographic and health system factors, with special reference to the practice of administering two or more doses of oxytocic medicines to augment labour, with stillbirths in two districts of Bihar.

## Methods

This study was conducted in two UNICEF priority districts of Bihar -Gaya and Purnea- between July and August 2015 using a case-control study design. In these two districts, 23 blocks were chosen (16 from Gaya and 7 from Purnea) based on the number of stillbirths reported in HMIS from facilities. In order to minimize the extent of recall bias, the enrolment of participants was limited to the previous six calendar months.

### Sample size

The sample size was calculated for a 1:2 case-control study, with the assumption that the prevalence of the principal exposure (administration of more than two doses of medicines to augment labour) is 20% among controls, power of 80%, an estimated odds ratio (OR) depicting the strength of association between exposure and stillbirth of 1.5, and the level of significance being 0.05. The estimated sample size was 388 cases and 776 controls. This was rounded off to 400 cases and 800 controls.

### Study participants

Definitions used to report cases of stillbirths vary across the country. For our study, we identified a case as “any baby born dead after the 24th week of pregnancy” [18]. “Born dead” included babies that did not breathe or cry or show other signs of life. Both singleton and multiple pregnancies were included. For selection of cases, the line lists of all facility-based stillbirths of the past 6 months were obtained from HMIS from the selected blocks. The interviewers then contacted the frontline workers of the respective villages/wards within these blocks to trace the mothers/families of these stillborn babies. Mothers/families not willing to participate were excluded from the study. For each included case, two controls were identified. These controls were two live born babies from the same village/ward as the case, born just before, at the same time or just after the case.

### Data collection

A participant information sheet and consent form were developed for the study. A short screening tool was developed to differentiate stillbirths from early neonatal deaths. A validated World Health Organization (WHO) verbal autopsy semi-structured tool, adapted in our previous study, was used to collect data on risk factors [18, 19] [see Additional file 1]. Some context and region-specific factors were added, and pre-tested in the field before initiating data collection.

Trained interviewers visited the families of the stillbirths with the help of the information provided in the line lists, confirmed the cases of stillbirths based on the screening tool and obtained informed consent. For the confirmed stillbirths, controls were selected from the same area. The interviews took place at the homes of the participants and lasted for more than half an hour each. Data were collected on paper forms by giving a unique identification number, and later used for data entry and analyses. The data were collected by interviewing the mother and family members. Medical records were used wherever they were made available, especially for labour and delivery related information.

### Risk factors

Risk factors mentioned in the literature and those listed in the WHO tool were included in the study. It is a reported common practice to administer oxytocics (usually oxytocin) during labour to the pregnant woman for augmentation [15–17]. The primary exposure considered was “administration of two or more doses of oxytocics during labour” before reaching the health facilities for delivery. Since the responses were obtained from the mothers, the term “medicines” was used as a proxy term for oxytocics. Mothers generally being unaware of the name of the medicines, we tried to confirm the intake of oxytocics by asking about the route of administration. Responses obtained as ‘injections’ were considered as proxy for oxytocin and those obtained as ‘oral’ were assumed to be misoprostol. Maternal factors including complications during pregnancy and delivery such as: history of bleeding during pregnancy or before delivery (ante-partum haemorrhage), a record of high blood pressure or a history given by the mother (pre-eclampsia), intake of drugs other than iron and calcium during pregnancy, exposure to active or passive smoking, and physical injury and violence as reported by mothers. Fetal factors included prematurity (assessed from gestational age in weeks calculated from last menstrual period, verified from records or elicited from history in the absence of records), low birth weight if records were available, and congenital malformations elicited from history. Information pertaining to approximate time taken to reach the facility for institutional deliveries, mode of transport and time taken to initiate management was reported by mothers/family members.

The modifiable health-system related risk factors included in the study were: less than the recommended number of antenatal-care visits (3) that can facilitate early identification of complications and timely management, administration of more than two doses of oxytocics to augment labour before reaching the health facilities, complications during labour that can be addressed with safe and skilled delivery, delay in reaching a facility, means of transport to reach a facility, and a delay in attending to the pregnant woman after reaching the facility.

### Data management

A database was designed in Census and Survey Processing System (CS pro version 5.0, 2012). Data management included data entry, creation of data description tables, validation of database, cleaning, processing and analyses of data. Data were maintained in password-protected files and patient confidentiality was maintained at all times. Data access was restricted to the research team only.

### Statistical analyses

Statistical Package for the Social Sciences (SPSS) version 19.0, 2010 was used for the quantitative analyses. A conceptual framework was developed to guide the analyses in which risk factors were categorized into four hierarchical levels [17]:

i) the sociodemographic factors such as age of the mother and father, their education, occupation were considered as the most distal determinants;
ii) antenatal factors such as birth order, gestation period, history of previous stillbirths, early registration of pregnancy, number of antenatal visits, iron supplementation, ultrasonography, exposure of active or passive smoking, history of injury, accidents and physical violence, medical conditions like diabetes and intake of indigenous medicines during pregnancy were considered as the intermediate determinants;
iii) factors during the last trimester of pregnancy such as vaginal bleeding, foul smelling discharge, burning micturition, oedema, severe or persistent abdominal pain, severe anemia requiring blood transfusion, high blood pressure were the proximate factors;
iv) labour and childbirth-related factors such as administration of 2 or more doses of oxytocics to augment labour, any complication during labour (such as convulsions, excessive bleeding, fever, high or low blood pressure, obstructed labour, excessive perspiration, blurring of vision), mode of transport to reach facilities, time taken to reach the facility and time taken to attend to the women after reaching the facilities.

Every factor with at least five respondents in each category was examined for association with the outcome of interest (live birth/stillbirth) using bivariate analyses. At every step in the analyses, *p* < 0.05 was considered as the cut-off for statistical significance. For multivariable analyses, a hierarchical approach was used [20]. From Model 1 through to Model 4, we considered distal factors and moved to proximal ones in successive steps. The factors that were statistically significant or biologically plausible in one model were considered in the successive model, starting from sociodemographic factors (Model 1), antenatal factors (Model 2), factors related to the third trimester of pregnancy (Model 3), followed by delivery related factors in Model 4. Model 4 was the final multivariable regression analyses model.

Unadjusted and adjusted odds ratios (AOR) were calculated for the risk factors. Attributable fractions (AF) and population attributable fractions (PAF) were calculated for modifiable health system risk factors.

### Quality assurance measures

Permission was sought from the State and district authorities at the outset. We obtained approval from Institutional Ethics Committee of Indian Institute of Public Health, Delhi, Public Health Foundation of India. Potentially eligible parents were given detailed information about the study in local language by the project team prior to data collection. Written consent was taken from every mother/family member who voluntarily agreed to participate in the study.

## Results

The interviewers screened a total of 421 cases of stillbirths prior to the interviews, from both districts based on the line lists available so as to meet the sample size. Cases were confirmed as stillbirths (based on signs of life immediately after birth) during the screening; it was observed that 21 (5%) listed stillbirths were actually early neonatal deaths, falsely reported as stillbirths. These were excluded from the study. Thus, data were collected from 400 confirmed stillbirths and 800 controls.

### Socio-demographic variables

The cases and controls, though comparable, were statistically significantly different with regards to maternal age, occupation of mothers, education and occupation of fathers (Table 1).

**Table 1:** Socio demographic profile of cases and controls

Socio demographic variables	Cases n (%) *N* = 400	Controls n (%) *N* = 800	*p*-value^*^
Age of father (mean) in years	27.32 (4.2)	27.5 (3.2)	0.26
Age of mother (mean) in years	23.9 (3.7)	24.5 (3.1)	0.002^*^
Education of mothers			0.78
Illiterate	292 (73.0%)	563 (70.4%)	
Just literate	66 (16.5%)	126 (15.8%)	
Primary school completed	23 (5.8%)	59 (7.4%)	
High and senior secondary school completed	18 (4.5%)	49 (6.1%)	
Graduation and above	1(0.3)	3 (0.4)	
Occupation of mothers			0.016^*^
Agriculture	31 (7.8)	105 (13.2)	
Home maker	283 (70.8)	517 (64.6)	
Office job	0	2 (0.3)	
Manual laborer	84 (21.0)	175 (21.9)	
Others	2 (0.5)	1 (0.1)	
Education of fathers			<0.001^*^
Illiterate	152 (38.0)	310 (38.8)	
Just literate	120 (30.0)	207 (25.8)	
Primary school completed	77 (19.3)	100 (12.5)	
High and senior secondary school completed	44 (11.1)	162 (20.3)	
Graduation and above	7 (1.8)	21.0 (2.6)	
Occupation of fathers			0.023*
Agriculture	127 (31.8)	248 (31.0)	
Office job	8 (2.0)	26 (3.3)	
Industry	24 (6.0)	85 (10.7)	
Manual laborer	237 (59.3)	435 (54.4)	
Others	4 (1.1)	6 (0.7)	

*Values of *p* < 0.05 considered as statistically significant difference or association, based on chi-square estimates for proportions and *t*-test for means

93% (372/400) mothers had registered with health facilities during pregnancy. However, the proportions of first trimester registrations were 47% and 60% among cases and controls respectively. Also, a significantly higher proportion of mothers of controls had completed three or more antenatal visits (53.5% v/s 35.3% among cases). 1.3% of the stillbirths in the study were home deliveries, but had been referred to facilities subsequently. Among controls, 22.5% were born at home.

### Risk factors

Of all the factors, 22 differed between cases and controls on bivariate analyses (*p* < 0.05) (Table 2). History of augmentation of labour with two or more doses of oxytocics was significantly different between cases and controls (36.3% v/s 26.1%; *p* < 0.001).

**Table 2:** Risk factors for stillbirths among cases and controls – bivariate analyses

Risk factors	Cases n (%) *n* = 400	Controls n (%) *n* = 800	*p*-value^*^
Sociodemographic factors			
Age of the mother >30 years	23 (5.8)	15 (1.9)	<0.001^*^
Occupation of mother (working outside home)	117 (29.3)	283 (35.4)	0.03^*^
Education of father (illiterate)	152 (38.0)	310 (38.8)	0.8
Occupation of father (non-manual labourer)	163 (40.8)	365 (45.6)	0.11
Sex of the child (male)^a^	218/393 (55.4)	485/800 (61.1)	
Antenatal factors			
Birth order			
Primigravidae	174 (43.5)	275 (34.4)	<0.001^*^
Multigravidae	226 (56.5)	525 (65.6)	
Gestation period (*n* = 1178)			
< 28 weeks	0	3 (0.4)	<0.001^*^
< 37 weeks	298 (75.3)	286 (36.7)	
Mean interval between present and the last pregnancies among multigravida (months)	20.1 (10.3)	20.2 (9.4)	0.9
Any previous stillbirths among multigravida mothers	33 (14.6)	59 (11.3)	0.2
Registered with health facility during pregnancy	372 (93.0)	754 (94.3)	0.39
Proportion of women registered in 1st trimester	187 (46.8)	479 (59.9)	<0.001^*^
Received 3 or more ANC checkups	141 (35.3)	428 (53.5)	<0.001^*^
Intake of iron tablets during pregnancy	263 (65.8)	567 (70.9)	0.07
Multiple pregnancy (current pregnancy)	4 (1.0)	6 (0.8)	0.65
Tetanus toxoid during pregnancy	385 (96.3)	772 (96.5)	0.83
Ultrasound during pregnancy	104 (26)	205 (25.6)	0.9
Exposure to active or passive smoking	156 (39)	170 (21.3)	<0.001^*^
History of chewing tobacco during pregnancy	89 (22.3)	59 (7.4)	<0.001^*^
History of injury or accident during pregnancy	41 (10.3)	11 (1.4)	<0.001^*^
History of any physical violence during pregnancy	50 (12.5)	56 (7.0)	0.002^*^
Gestational diabetes	12 (3.0)	30 (3.8)	0.5
Intake of indigenous medicines during pregnancy	25 (6.3)	33 (4.1)	0.44
Factors during last trimester of pregnancy			
Bleeding per vagina during pregnancy	146 (36.5)	106 (13.3)	<0.001^*^
Excessive or foul smelling vaginal discharge during pregnancy	75 (18.8)	62 (7.8)	<0.001^*^
Burning micturition	199 (49.8)	312 (39.0)	<0.001^*^
Oedema (hand or face or legs)	225 (56.3)	317 (39.6)	<0.001^*^
Severe or persistent abdominal pain during pregnancy (not labour pain)	131 (32.8)	211 (26.4)	0.02^*^
Blurring of vision or severe headache during pregnancy	132 (33.0)	123 (15.4)	<0.001^*^
Severe anemia requiring blood transfusion	19 (4.8)	26 (3.3)	0.2
High blood pressure during pregnancy	46 (11.5)	21 (2.6)	<0.001^*^
Factors related to labour and childbirth			
Any complication during labour	140 (35.0)	152 (19.0)	<0.001^*^
Administration of 2 or more doses of medicines to augment labour	145 (36.3)	208 (26.1)	<0.001^*^
Duration of labour (12 h or more)	37 (9.3)	71 (8.9)	0.53
Mode of transport to reach facilities	*n* = 395	*n* = 616	<0.001^*^
Ambulance	93 (23.5)	148 (23.9)	
Private vehicle	200 (50.6)	374 (60.4)	
Any public transport	102 (25.8)	94 (15.0)	
Time taken to reach facility	*n* = 393	*n* = 616	
Up to 30 min	253 (64.4)	439 (71.3)	0.02^*^
> 30 min	140 (35.6)	177 (28.7)	
Time to attend after reaching the facility	*n* = 392	*n* = 615	
Up to 10 min.	171 (43.5)	390 (63.4)	<0.001^*^
> 10 min.	222 (56.5)	225 (36.6)	
Mode of delivery^b^			
Vaginal (spontaneously or with little maneuver)	375 (93.7)	783 (98.1)	
Instrumental delivery	24 (6.0)	10 (1.3)	
Caesarean section	1 (0.3)	5 (0.6)	
Presence of any visible structural birth defect^b^			
Yes	3 (0.8%)	3 (0.4%)	
No	350 (87.5%)	782 (97.8%)	
Do not know	47 (11.8%)	15 (1.9%)	

^*^Values of *p* < 0.05 considered as statistically significant difference or association, based on chi-square estimates for proportions

^a^Sex of stillborn children was not reliable hence was not considered for analyses

^b^Because the cell size was less than 5, these were not included in the analyses

A multivariable model was developed using a hierarchical approach with factors that had different distribution in cases and controls in bivariate analyses (*p* < 0.05). The final model (Model 4) explained 35% (Nagelkerke R^2^) of the variance in the stillbirths and correctly classified 76.1% of cases.

Of all the risk factors examined, the final model included maternal age (more than 30 years), primigravidae, preterm labour (<37 weeks), antenatal factors such as exposure to active or passive smoking, history of injury or accident, history of physical violence, bleeding per vagina, oedema, blurring of vision or severe headache and high blood pressure and intranatal factors such as complications during labour and time taken to attend to the pregnant woman after reaching the facility. Since blurring of vision, oedema and hypertension could be biologically related, we checked for collinearity, which was found to be poor, hence these variables were considered as independent variables for the analyses. Of these factors, the modifiable health system-related risk factors included longer than 10 min taken to attend to women after they reach the facilities (AOR 3.6; 95% CI 2.5–5.1), untreated hypertension during pregnancy (AOR 2.9; 95% CI 1.5–5.6) any complication during labour, warranting treatment (AOR 1.7; 95% CI 1.2–2.4). Administration of two or more doses of oxytocics to augment labour was associated with stillbirths (Unadjusted OR 1.6; 95% CI 1.2–2.1). However, the multivariable analyses failed to detect an association between the two factors (AOR 0.98, 95% CI 0.7–1.4) (Table 3).

**Table 3:** Multivariable analysis of risk factors for stillbirths using hierarchical approach

Risk factors	Model 1	Model 2	Model 3	Model 4
Sociodemographic factors				
Age of the mother >30 years	3.2 (1.6–6.2)	3.8 (1.2–8.0)	3.5 (1.7–7.5)	4.3 (1.7–10.7)
Occupation of mother (working outside home)		–	***–***	***–***
Antenatal factors				
Birth order (primigravidae)		1.7 (1.3–2.3)	1.6 (1.2–2.2)	1.8 (1.3–2.6)
Preterm labour (<37 weeks)		4.8 (3.6–6.5)	4.0 (2.9–5.5)	4.5 (3.2–6.5)
Registration of pregnancy in first trimester		0.5 (0.4–0.7)	0.6 (0.4–0.8)	–
Received 3 or more ANC checkups		0.8 (0.6–1.0)	1.0 (0.7–1.4)	–
Exposure to active or passive smoking		1.9 (1.4–2.6)	2.3 (1.7–3.3)	2.1 (1.4–3.0)
History of chewing tobacco during pregnancy		1.6 (1.0–2.5)	1.5 (0.9–2.3)	–
History of injury or accident during pregnancy		4.3 (2.0–9.3)	4.3 (2.0–9.5)	10.7 (3.4–33.7)
History of any physical violence during pregnancy		2.0 (1.2–3.3)	2.3 (1.4–3.9)	2.5 (1.4–4.6)
Factors during last trimester of pregnancy				
Bleeding per vagina during pregnancy			2.2 (1.5–3.2)	2.6 (1.7–3.8)
Excessive or foul smelling vaginal discharge during pregnancy			–	–
Burning micturition			1.4 (1.0–1.9)	–
Oedema (hand or face or legs)			1.4 (1.1–1.9)	1.8 (1.3–2.6)
Severe or persistent abdominal pain during pregnancy (not labour pain)			–	–
Blurring of vision or severe headache during pregnancy			1.7 (1.2–2.5)	1.9 (1.3–2.8)
High blood pressure during pregnancy			3.1 (1.6–5.8)	2.9 (1.5–5.6)
Factors related to labour and childbirth				
Any complication during labour^a^				1.7 (1.2–2.4)
Administration of 2 or more doses of medicines to augment labour				–
Time take to reach facility (>30 mins)				–
More than 10 mins to attend after reaching the facility				3.6 (2.5–5.1)

^a^Complication during labour include convulsions, excessive bleeding, fever, high or low blood pressure, obstructed labour, excessive perspiration, blurring vision and kidney failure

Among mothers of cases and controls who reported having some complications during labour, significant differences were observed in time taken to reach the facility (2nd delay^1^; 33.5 min v/s 25 min, *p* < 0.001) and time taken to initiate management (3rd delay^1^; 15.5 v/s 9 min, *p* < 0.001). Our analyses indicated that women who had stillbirths were almost 4 times more likely to have been attended to after 10 min of reaching the facilities for delivery.

Apart from medical factors, some social factors predisposed the pregnant women to stillbirths. Mothers of stillborn children were 2.5 times more likely to have been a victim of physical violence during pregnancy, as compared to mothers of controls. Exposure to active or passive smoking almost doubled the chances of stillbirth.

AF for any complication during labour was 0.56 (95% CI 0.42–0.67), more than 30 min to reach the facility was 0.48 (95% CI 0.31–0.60) and initiation of management 10 min after reaching the facility was 0.68 (95% CI 0.58–0.75). Reaching a facility within 30 min, initiation of management within 10 min of reaching the facility and timely management of complications during labour could have prevented 17%, 37% and 20% of stillbirths respectively.

## Discussion

In the present population-based case-control study, untreated hypertension during pregnancy, longer than 10 min taken to attend to pregnant women when they reach the facilities for delivery and presence of any complication during labour that warrants treatment emerged as significant modifiable health system risk factors for stillbirths. The other risk factors included increased maternal age, preterm births, primigravidae, exposure to active or passive smoking, and history of injury or physical violence during pregnancy.

Stillbirths is a sensitive marker of the strength of the health system [21]. In Indian settings, stillbirths have customarily been under-reported, given the lack of consistency in the definitions and lack of demand for data surrounding stillbirths [22]. Additionally, early neonatal deaths are falsely reported as stillbirths; in a study conducted elsewhere in India it was found to be 15% [18]. Irrespective of settings, the largest proportions of stillbirths are antepartum deaths [23]. In India, the current reporting system for stillbirths does not include their categorization as fresh or macerated. As a result, it is difficult to differentiate the antepartum stillbirths from intrapartum stillbirths. Moreover, due to the retrospective nature of our study design, it was difficult to elicit whether the stillbirths were antepartum or intrapartum, hence they were considered as one group in this study.

Identification of high-risk pregnancies during antenatal period can facilitate timely initiation of therapy and referral to the appropriate facility to prevent or minimise antepartum deaths [24]. In our study, the coverage of antenatal care was less than 60% even among live births, thereby plummeting the chances of early identification and management of high-risk pregnancies. It is suggested that four antenatal visits may not positively impact the outcome of pregnancy until the coverage is at least 60% [21]. Women with high blood pressure in pregnancy were almost three times more likely to have stillbirths. In our study, 52% of women with high blood pressure among cases reported having consumed antihypertensive medications while it was 62% among controls, although compliance to advice and medication would be a concern in both groups. Similar findings have been reported from other studies [25, 26] suggesting that early diagnosis and adequate medical intervention for hypertensive disorders can help reduce the risk of stillbirths.

Exposure to active or passive smoking led to an almost 2-fold risk of having a stillborn baby in our study, similar to existing evidence that maternal smoking was associated with a 47% increase in the odds of stillbirth [27]. Mothers of stillbirths were more likely (*p* = 0.002) to have been the victims of physical violence during pregnancy, which was similar to other studies [28, 29]. Since this information is quite sensitive in nature, there is a likelihood of under-reporting [30]. This may have resulted in a non-differential misclassification of the exposure, meaning that the actual strength of association might be more than what is reported in this study.

The optimal caesarean section (CS) rate to reduce the proportion of intra-partum stillbirths lie between 5 and 10% [31]. Evidence from LMICs shows that with every percentage rise in CS rate from 0 to 8%, intra-partum stillbirth drops by 1.6 per 1000 births [31]. The rate of CS in our study was <1%, far below the optimal rates. Studies have also demonstrated that good quality obstetric care, and not mere provision of CS services, is critical [32]. In a recent analysis, the maximum benefit was noted from appropriate care during labour and childbirth, including complications (70% of all stillbirths averted), followed by enhanced antenatal care focussing on detection of complications (21%) [33]. The proportions of instrumental deliveries among the cases and controls in our study were 6% and 1.3% respectively. There may be instances in which instruments might have been applied to expedite the delivery in a confirmed case of stillbirth. Hence, it is difficult to interpret the association between instrumental deliveries and stillbirths.

In our study, mothers who delivered prematurely were 4.5 times more likely to have a stillborn child, as compared to full-term deliveries. Preterm births and low birth weight babies increase the risk of stillbirths manifold [34]. A study highlighted that spontaneous preterm birth or preterm ruptured membranes were the key factors in 41% of stillbirths less than 28 gestational weeks [35]. This is in sharp contrast to our study where more than 75% stillbirths occurred between 28 and 37 weeks of gestation, although we took 24 weeks as the cut-off to identify stillbirths. However, it is not known how many stillbirths could have been averted through public health interventions like administration of antibiotics for preterm, premature rupture of membranes and antenatal corticosteroids for preterm labour [36].

In our study, administration of two or more doses of oxytocics to augment labour was associated with stillbirths in the unadjusted analyses. More than 35% mothers of stillborn children and 25% mothers of live born babies reported the use of such medications before reaching the facilities. Previous studies from India have shown that unwarranted use of oxytocics increases the risk of stillbirth, similar to our finding when considered independently [14, 16, 17, 37]. Oxytocics are usually administered only for a valid indication in facilities with provision for C-Sections [38]. Use of such oxytocics only to hasten the process of delivery without a valid indication and medical supervision may be detrimental. Nevertheless, rational use of oxytocics in health facilities along with regular intrapartum monitoring can positively favour obstetric outcomes.

Substandard obstetric care contributes to 20–30% of all stillbirths [35]. Reaching a facility within 30 min, initiation of management within 10 min of reaching the facility and timely management of complications during labour could have prevented 17%, 37% and 20% of stillbirths respectively, according to our analyses. It is comparable to the estimated reduction in stillbirths by 25% with 60% coverage of basic interventions for skilled obstetric care [36]. This is however true only for prevention of intra-partum stillbirths. In our study, nearly 21% of mothers of cases and 48% mothers among controls admitted that they felt the last fetal movement during labour implying that more cases could have been antepartum stillbirths.

The study had its own set of limitations. Birth weight is an important parameter, since management of fetal growth restriction can avert 20% of antepartum and intra-partum stillbirths [36]. However, weighing of stillborn babies is not yet practiced in India [18]. Stillbirth reporting has improved through routine HMIS, however recording of all birth outcomes using comparable definitions, ante-partum versus intra-partum stillbirths and birth weight are weak links within the system [29, 34]. This was observed in our study as well. Increased and improved data availability can provide a solution for targeted interventions where those would be most effective [1]. Also, since the cases in our study were chosen from lists available at healthcare facilities, those women who had a stillbirth at home and who never visited the facilities for any post-partum complications were missed out completely, since they would not be registered in the HMIS. This was a major limitation in a situation where one-third to one-fourth of the deliveries take place at home. In the absence of reliable data, we were not certain if home-delivered stillbirths are different from facility-based stillbirths. Home-based stillbirths may be less likely to exhibit complications compared to facility stillbirths. There is a likelihood of bias resulting due to differential misclassification, which can change the direction of the observed association. The sample size was calculated considering administration of more than two doses of oxytocics to augment labour as the principal exposure. For the remaining factors, exploratory analysis was conducted and hence may not have been adequately powered to establish an association.

Besides these, our inability to differentiate between antepartum and intrapartum stillbirths remains a major challenge. Since we are focussing on health system related factors, it would have been useful to analyse the two groups separately. Paucity of this information might have led to undermining of the actual effect. From the reported information on the instrumental deliveries, it is difficult to ascertain if instruments were used more often to expel stillborn babies or these were used to deliver live babies who were later declared as stillborns when the babies were found dead. Approximate time to reach the facilities and time to initiate management after reaching the facilities were self-reported by mothers/family members. This could have resulted in recall bias; we tried to minimize this bias by keeping the interval between the event and the interview to less than 6 months. However, this might have affected both the groups leading to non-differential misclassification and hence the actual strength of association could be more than what is inferred from this study.

Since autopsies or other tests were not performed on stillborn babies, it is difficult to establish any causation. This poses a hindrance to understanding the cause of death, and increases the proportion of unexplained deaths [35]. Use of a validated verbal autopsy tool to collect data could address this lacuna to some extent. The tool has reasonable validity in identifying and discriminating between causes of stillbirth [19, 39, 40]. Any history of medical complication during pregnancy in our study was corroborated from available records. In our setting, availability of medical records was a challenge, thus resulting in probable reporting bias. However, our study focussed on risk factors and not causes of stillbirths. Besides, this study though representative of the state, focussed mainly on rural populations.

Despite these limitations, the study highlights the importance of basic and advanced antenatal and intra-natal care for averting stillbirths. Conducted on a large sample and in household settings, the study captured existing factors related to obstetric care. This work is one of the few exploratory studies which have used routine health information system data to identify stillbirths from the community in this setting. Yet another strength of this study was the ability to relate the health-system related risk factors with stillbirths.

## Conclusions

To conclude, a strong health system with a good coverage of basic antenatal care and timely institution of emergency obstetric services can avert a significant proportion of stillbirths in similar settings. A good quality antenatal care package can improve compliance, and detect complications early during pregnancy for timely action. Certain unwarranted complications that may occur during the last trimester of pregnancy can also be adequately addressed with appropriate measures taken as soon as women reach the health facility. Stillbirths are adverse pregnancy outcomes, most of which can be averted by a pro-active and strong health system through appropriate obstetric practices. Judicious use of oxytocics and other medications during labour can improve pregnancy outcomes. Additionally, mechanisms to improve routine surveillance of stillbirths to detect contributory factors and the reporting of stillbirths including information on birth weight and fresh vs. macerated stillbirth would contribute to strengthening the health information system.

## Additional file

Additional file 1: Verbal Autopsy tool. Description: Used for collecting data related to stillbirths from mothers. (PDF 384 kb)

## Abbreviations

AF: Attributable Fraction
AOR: Adjusted Odds Ratio
CS: Caesarean Section
HMIS: Health Management Information System
ICD-10: The International Classification of Diseases, 10th revision
LMIC: Low and Middle Income countries
OR: Odds Ratio
PAF: Population Attributable Fraction
PHFI: Public Health Foundation of India
RR: Relative Risk
SBR: Still Birth Rate
SRS: Sample Registration System

## Footnotes

1 Time taken for a pregnant woman to reach a facility for delivery is termed as 2nd delay and time taken to initiate management after she reaches a facility is termed as 3rd delay. These are part of the 3 delay model which identifies three groups of factors affecting women accessing safe maternal healthcare.
