# Supplementary material for: Risk factors for stillbirths: how much can a responsive health system prevent?
Source: BMC Pregnancy Childbirth. 2018 Jan 18;18:33. doi: 10.1186/s12884-018-1660-1 (PMC5774063; doi:10.1186/s12884-018-1660-1)
Supplement: Additional file 1: — Verbal Autopsy tool. Description: Used for collecting data related to stillbirths from mothers. (PDF 384 kb) [file 12884_2018_1660_MOESM1_ESM.pdf]

VERBAL AUTOPSY FOR ASCERTAINING THE RISK FACTORS FOR STILLBIRTHS

Indian Institute of Public Health Delhi, Public Health Foundation of India

Participant Code

District code	1- case<br>2-control<br>3- control	Number of the child

Date of survey

D	D	M	M	Y	Y

**1. Information of the child**			
1.1	Name of the child, if kept?		
1.2	Address		
**2. Background Information**		**Mother**	**Father**
2.1	Full Name		
2.2	Age (in completed years)		
2.3	Date of Birth (DD/MM/YY) – if known		
**3. Socio economic history**		**Mother**	**Father**
3.1	What is your educational qualification<br>1. Illiterate (Cannot read or write)<br>2. Literate (Can read and write, but not entered school)<br>3. Primary school completed<br>4. High school completed<br>5. Senior secondary<br>6. Graduation<br>7. Post graduation & above	3.1.1	3.1.2
3.2	What is your occupation?<br>1. Agriculture<br>2. Office Job<br>3. Industry<br>4. Manual Laborer<br>5. House wife<br>6. Others (specify)____	3.2.1	3.2.2
3.3	If Code 3 (working in industry), specify the type of industry	3.3.1	3.3..2
3.4	What is your annual income of the household?<br>1. Less than 1 lakh<br>2. 1 - 3 lakh<br>3. 3.1 - 5 lakh<br>4. More than 5 lakhs<br>5. Don't Know		

4.Open history questions

Instructions to interviewer: Allow the respondent to tell you about the pregnancy, delivery and the baby's illness in her own words. Write down what the respondent tells you in her own words. Do not prompt except for asking whether there was anything else after the respondent finishes. Keep prompting until the respondent says there was nothing else. While recording, underline any unfamiliar terms.

For stillbirths (i.e., no cry, no breathing, no movement at birth), ask: Could you tell me about the pregnancy for this baby, labour and delivery, what the baby was like at birth and what happened after the delivery?

Prompt "Was there anything else"

	5. Obstetric History				
**5.1**	**Antenatal care**				
5.1.1	Registered with the health facility during pregnancy	Yes – 1, No – 2			
5.1.2	In which trimester, the pregnancy was registered?<br>1. First trimester (0-3 months)<br>2. Second trimester (4-6 months)<br>3. Third trimester (7-9 months)				
5.1.3	Did you receive 3 or more ante-natal checkups	Yes – 1, No – 2			
5.1.3.1	When was last ANC done? (in which month of pregnancy)				
5.1.4	Did you take any of the following supplements during this pregnancy?	Iron Folic acid (IFA)	Yes – 1, No – 2	Tablets Received (No.)	Tablets Consumed (No.)
		Calcium	Yes – 1, No – 2		
5.1.5	Tetanus Toxoid During pregnancy	Yes – 1, No – 2			
5.1.6	Ultrasound during pregnancy	Yes – 1, No – 2			
5.1.6.1	If yes, any specific indication for ultrasound during pregnancy? (if multiple ultrasounds done, specify indication for each separately)				
**5.2**	**Personal History**				
5.2.1	Age at the time of conception of this pregnancy				
5.2.2	Birth order of this pregnancy				
5.2.3	Birth interval between the last two pregnancies (in months)				
5.2.4	No. of children (whether livebirth or stillbirth) – (information to be obtained in chronological order of the children born)	**S No.**	**Outcome**	**Sex**	**Present age**
5.2.5	What was the duration of last pregnancy (in weeks)	Yes-1, No-2			
5.2.6	Plurality of pregnancy	Yes-1, No-2			
5.2.7	Previous history of still birth	Yes-1, No-2			
5.2.8	History of exposure to active or passive smoking (*biddi, cigarette, hookah etc.)*	Yes-1, No-2			
5.2.8.1	Did you chew tobacco products (*zarda, khaini, paan, gutka, gul, tambakhoo etc.) during pregnancy?*	Yes-1, No-2			
5.2.9	History of Injury/accident anytime during pregnancy	Yes-1, No-2			

5.2.10	History of any physical violence anytime during pregnancy	Yes-1, No-2
5.2.11	History of any illness at any time during the pregnancy<br>*(Multiple responses are possible)*<br>1. Diabetes<br>2. Hypertension<br>3. Epilepsy<br>4. Thyroid disease<br>5. Heart Disease<br>6. Allergy<br>7. TB<br>8. HIV<br>9. Fever with rash<br>10. Others (specify)	
**5.3**	**Medical and Drug History**	
5.3.1	Did you take any drug during pregnancy?<br>*(Multiple responses are possible)*<br>1- Anti-epileptics<br>2- Anti-diabetics<br>3- Anti-hypertensive<br>4- Analgesic/ Pain killer<br>5- Any tablets from other sources (specify)<br>6- None	
5.3.1.1	If yes, specify the time period (trimester)<br>*(Multiple responses are possible)*<br>1- Months 0-3<br>2- Months 4-6<br>3- Months 7-9	
5.3.2	Did you take any indigenous medication during pregnancy?	Yes ,as informed by mother-1,Yes, as informed by other source-2, Previous pregnancy-3,No-4
	If 'No', go to question no. 5.4	
5.3.2.1	If yes, specify the time period<br>*(Multiple responses are possible)*<br>1- Before conception<br>2- Month 1<br>3- Month 2<br>4- Month 3<br>5- Months 4-6<br>6- Months 7-9	
5.3.2.2	Where was the drug procured from?<br>1- Allopathic Doctor<br>2- AYUSH Doctor<br>3- Quack<br>4- Pharmacy<br>5- Religious Leaders<br>6- Others (specify)_____________	
5.3.2.3	What was reason behind taking these medicines? (e.g. male child, fair child, smooth labour etc)	
**5.4**	**Problems during last trimester of pregnancy**	

5.4.1	Bleeding from vagina	Yes-1, No-2
5.4.2	Smelly or excessive vaginal discharge	Yes-1, No-2
5.4.3	Burning micturition	Yes-1, No-2
5.4.4	Hand or face or leg swelling	Yes-1, No-2
5.4.5	Severe or persistent abdominal pain that was not labour pain	Yes-1, No-2
5.4.6	Blurring of vision or severe headache	Yes-1, No-2
5.4.7	Convulsions	Yes-1, No-2
5.4.8	Severe anemia requiring blood transfusion	Yes-1, No-2
5.4.9	High blood pressure	Yes-1, No-2
5.4.10	Diabetes	Yes-1, No-2
5.4.11	Fever	Yes-1, No-2
	Any other (specify)	Yes-1, No-2
	**6. Before labour**	
6.1	When did the baby last move in the womb?<br>1- During labour or delivery<br>2- 1-2 days before delivery<br>3- 3-6 days before delivery<br>4- A week or more before delivery<br>5- Don't Know<br>6- Never moved	
	**7. Labour and Delivery**	
7.1	Where was the baby delivered?<br>1- Government Hospital<br>2- Private Hospital<br>3- On route to hospital<br>4- Home<br>5- Others (specify)______	
7.2	Which mode of transportation you used to reach the health facility?<br>1- Ambulance<br>2- Private vehicle<br>3- Any public transport<br>4- Others, specify	
7.3	How this mode of transport was called for<br>1- Self payment<br>2- Payment through JSSK	
7.4	How far was the health facility where the delivery happened?<br>1- Within 5 kms<br>2- 5-10 kms<br>3- 10-15 kms<br>4- More than 15 kms	
7.5	How much time did you take to reach the facility? (in minutes)	

7.6	How much time did the first healthcare provider take to attend to you after you reached the hospital? (in minutes)	
7.6.1	Who was the healthcare provider?<br>1- Doctor<br>2- Nurse/ANM<br>3- Dai<br>4- ASHA<br>5- Other (specify)<br>6- Don't know	
7.7	Were you referred from that hospital to some other hospital?<br>If yes, how many times were you referred to a different health facility?<br>1. Once<br>2. Twice<br>3. More than twice	Yes-1, No-2
	[If **No,** then go to Q. No**. 7.8]**	
7.7.1	If referred, how much time did you take to reach the place of delivery from the first health facility? (in minutes)	
7.7.2	How much time did the health care provider take to initiate the treatment after reaching the place of delivery? (in minutes)	
7.8	Who delivered the baby?<br>1- Doctor<br>2- Nurse/ANM<br>3- Dai<br>4- Relatives<br>5- Spontaneous/ unattended<br>6- Don't Know	
7.9	Was the labour….?<br>1- Spontaneous without medication<br>2- Induced with medication<br>3- Augmented with medication<br>4- No labour pains<br>5- Don't Know	
7.10	Any excessive dosage of medication (more than 2 doses) given before or during labour before reaching the facility?	Yes – 1, No – 2
7.10.1	If yes, was the excessive dosage of medication given for….<br>1. Augmentation of labour for a live birth<br>2. Expulsion of a dead fetus<br>3. Expulsion of a congenitally malformed baby<br>4. Do not know	
7.10.2	What was the drug/ form of drug?<br>(Tablet/ Injection/ liquid medication/ other_specify)	
7.10.3	Who gave that drug?<br>1- Doctor<br>2- Nurse/ANM	

	3- Dai/ MAMTA<br>4- Other (specify)	
7.10.4	What was the route of administration?<br>1- Injection<br>2- Tablet<br>3- Others(specify)	
7.11	When did the water break?<br>1- Before labour<br>2- During labour<br>3- Water never broke<br>4- Don't know	
7.12	Was the water foul-smelling?<br>1- Yes<br>2- No<br>3- Don't know<br>4- Water never broke	
7.13	Did any problem occur during labour<br>1- Yes<br>2- No<br>3- Don't know	Yes-1, No-2
7.13.1	If Yes, what?<br>1- Mother had convulsions<br>2- High blood pressure<br>3- Fever<br>4- Excessive bleeding<br>5- Cord around the child's neck<br>6- Others (Specify___________)<br>7- Not applicable (if 7.13 is 'No')	
7.14	How was the baby delivered<br>1- Normally through vagina<br>2- Baby was pulled out by manipulation by hand<br>3- Baby was pulled using an instrument (forceps/ vacuum)<br>4- C-Section<br>5- Do Not Know	
7.15	How much time did the labour and delivery take?<br>1- Less than 12 hours<br>2- Twelve hours or more<br>3- No labour pains<br>4- Don't know	
7.16	What were the treatment given at the time of delivery in labour room?<br>(multiple options separate with comma)<br>1- Oxytocin<br>2- Antibiotics<br>3- Magnesium sulphate<br>4- Mesoprostol<br>5- IV fluids<br>6- Oxygen<br>7- Blood transfusion<br>8- Other (Specify)	
	**8. After delivery**	

8.1	Did the baby cry immediately after birth?	Yes-1, No-2, NA-3 (for stillbirths)
	If 'Yes', go to question no. 8.2	
8.1.1	If no, what was done for the baby to cry?<br>1. Baby was made to cry in the<br>**A.** Labour room/ NBCC<br>I. Holding baby upside down<br>II. Tapping on the back of baby<br>III. NRP<br><br>**B.** Home (in case of home delivery) through<br>I. Holding baby upside down<br>II. Tapping on the back of baby<br>III. NRP	
8.2	How long did you stay in the hospital after your delivery?	
8.3	Did you receive any post-natal checkup?	Yes-1, No-2
8.3.1	If yes, how many home visits were made by the healthcare provider? (ASHA/ ANM)	
**9. Appearance of the baby after birth**		
9.1	Were there any bruises or marks on the baby's body at birth?<br>1- Yes<br>2- No<br>3- Don't know	
9.2	Did the baby suffer from any congenital defects?<br>1- Yes<br>2- No<br>3- Don't Know	
9.2.1	If yes, which defect?<br>1- Spinal cord defects<br>2- Big head (Hydrocephalus)<br>3- Cleft lip or palate<br>4- Umbilical stump defect (Oomphalocele)<br>5- Others (specify)_____________<br>6- Not applicable	
